# ErbB2 enhances mammary tumorigenesis, oncogene-independent recurrence and metastasis in a model of IGF-IR-mediated mammary tumorigenesis

**DOI:** 10.1186/1476-4598-9-235

**Published:** 2010-09-08

**Authors:** Craig I Campbell, James J Petrik, Roger A Moorehead

**Affiliations:** 1 University of Guelph, Dept of Biomedical sciences, 50 Stone Rd. E, N1G2W1, Guelph, ON, Canada

## Abstract

**Background:**

The type I insulin-like growth factor receptor (IGF-IR) and ErbB2 (Her-2) are receptor tyrosine kinases implicated in human breast cancer. Both proteins are currently the subject of targeted therapeutics that are used in the treatment of breast cancer or which are in clinical trials. The focus of this study was to utilize our inducible model of IGF-IR overexpression to explore the interaction of these two potent oncogenes.

**Results:**

ErbB2 was overexpressed in our RM11A cell line, a murine tumor cell line that overexpresses human IGF-IR in an inducible manner. ErbB2 conferred an accelerated tumor onset and increased tumor incidence after injection of RM11A cells into the mammary glands of syngeneic wild type mice. This was associated with increased proliferation immediately after tumor cell colonization of the mammary gland; however, this effect was lost after tumor establishment. ErbB2 overexpression also impaired the regression of established RM11A tumors following IGF-IR downregulation and enhanced their metastatic potential.

**Conclusion:**

This study has revealed that even in the presence of vast IGF-IR overexpression, a modest increase in ErbB2 can augment tumor establishment *in vivo*, mediate resistance to IGF-IR downregulation and facilitate metastasis. This supports the growing evidence suggesting a possible advantage of using IGF-IR and ErbB2-directed therapies concurrently in the treatment of breast cancer.

## Background

Receptor tyrosine kinases (RTKs) are transmembrane proteins with intracellular kinase domains that undergo phosphorylation in response to ligand binding. This group of proteins has a well established role in breast cancer, and thus many RTKs are currently the focus of directed therapeutics with a significant number of these therapies in clinical trials. Two such proteins with validated roles in breast cancer are ErbB2 (also known as Her2/neu), a member of the epidermal growth factor receptor family, and the type I insulin-like growth factor receptor (IGF-IR). A large amount of evidence implicating both in clinical breast cancer is emerging. In addition, both receptors have been validated as oncogenes through the generation and characterization of transgenic mouse models (reviewed in [1] and [2]).

The IGF-IR undergoes autophosphorylation on conserved intracellular tyrosine residues after binding its ligands IGF-I and IGF-II which subsequently triggers signal cascades involved in many processes including proliferation and evasion of apoptosis [3]. Common activated downstream tyrosine kinase cascades include the phosphatidyl inositol-3 kinase (PI-3K)/Akt and mitogen activated protein kinase (MAPK)/Erk1/2 pathways [4]. It is also widely accepted that the IGF-IR has the capacity to transform normal cells and its expression is required for transformation by other known oncogenes [5-8]. Studies have shown IGF-IR levels are highly expressed in 39-93% of human breast cancers [9]and in breast tumors expressing high levels of IGF-IR the receptor is expressed 10-14-fold higher on average compared to normal breast tissue [10,11]. In addition, phosphorylation of IGF-IR was reported to be 2-4-fold higher in breast cancer tissue, which translated to a 40-fold increase in active IGF-IR in some cases [11]. Regulation of IGF-IR expression in breast cancer appears quite complex as it has been associated with both a poor [12] and favorable prognosis [13] and may change depending on the stage of the tumor [14]. In mouse xenografts, syngeneic, and chemically induced models of mammary carcinogenesis, inhibition of the IGF-IR through a variety of means has yielded success in limiting tumor growth and proliferation [15-19]. In addition, the transforming potential of this protein has been confirmed with two transgenic mouse models, one using the native IGF-IR and the other employing a constitutively active form of the receptor [20,21]. In light of the mounting evidence suggesting a prominent role of the IGF-IR in breast cancer, numerous targeted therapies are currently in clinical trials [22,23].

Unlike the IGF-IR and other RTKs, ErbB2 has no known specific ligand [24]. However, it preferentially forms heterodimers with other members of the EGF receptor family, EGFR (ErbB1), ErbB3 and ErbB4 [25]. In addition, ErbB2 heterodimers were determined to enhance the mitogenic signal of ErbB1 and ErbB3 [26]. Gene amplification with subsequent overexpression of ErbB2 has also been shown to contribute to the formation of activated homodimers [27]. Downstream signaling cascades triggered by ErbB2 homo/heterodimers are very similar to those activated by IGF-IR. ErbB2 is overexpressed in 25-30% of human breast cancer cases and is correlated with poor prognosis and shorter disease free survival [28]. It has been established that human and mouse ErbB2 variants are capable of transforming normal murine mammary epithelial cells and NIH/3T3 fibroblasts [29,30]. As stated previously, a number of transgenic models have validated this observation; animals overexpressing wild type neu formed tumors with an average latency of 7 months while with an activated form of ErbB2, latency was decreased to 3 months [2]. Because of the clinical implications of ErbB2 overexpression and its transforming potential, a number of directed therapies have been developed for the treatment of breast cancer which target ErbB2 alone or in combination with other RTKs; one of which, trastuzumab (Herceptin) is currently used to treat Her2^+ ^breast cancer (reviewed in [31]).

There is a growing body of evidence suggesting an interaction between the IGF-IR and ErbB2 in clinical breast cancer. Different studies have shown a physical interaction between the two receptors through immunoprecipitation [32-34] and immunofluorescence co-localization staining [34]. It has also been determined that knocking down expression of IGF-IR in human breast cancer cell lines can attenuate ErbB2 phosphorylation; however, the reciprocal was not observed [34]. In addition, signaling though the IGF-IR has been shown to mitigate the growth inhibitory effects of trastuzumab on human breast cancer cells overexpressing ErbB2 [35]; in fact, it is becoming widely accepted that signaling through IGF-IR can contribute to resistance to ErbB2-directed therapies (reviewed in [36]), and it has been noted that a correlation between IGF-IR expression and trastuzumab resistance exists in clinical breast cancer cases [37]. The existence of IGF-IR/ErbB2 heterodimers has also been established and this association was shown to contribute to Herceptin resistance in human breast cancer cell lines; this interaction could be disrupted by treating with monoclonal antibody based therapy to IGF-IR [38]. Based on these findings, a number of groups have investigated different combinations of inhibitors of IGF-IR and ErbB2 signaling. Nordihydroguaiaretic acid (NDGA), a dual inhibitor of IGF-IR and ErbB2, was shown to promote cell death in trastuzumab resistant human breast cancer cell lines [39]. Also, a synergistic growth inhibitory relationship between trastuzumab and inhibitors of IGF signaling including small molecule inhibitors of IGF-IR, IGFBP-3 and a dominant negative IGF-IR has been established in ErbB2 overexpressing human breast cancer cell lines [40-42]. An IGF-IR inhibitor was even found to enhance ErbB2-mediated apoptosis in a human breast cancer cell line with very little IGF-IR expression; evidence of this inhibitor augmenting the suppression of ErbB2 phosphorylation was also discovered [32].

To examine the role of the IGF-IR in breast cancer, our lab has previously created a doxycycline-inducible transgenic mouse model (MTB-IGFIR). IGF-IR-induced transgenic animals develop multiple tumors with 100% penetrance and an average latency of approximately 50 d [21], with metastasis occurring in approximately 40% of mice (unpublished observations). From one such primary tumor, the RM11A cell line was established and was shown to maintain doxycycline-inducible overexpression of the IGF-IR [43]. Enhanced phosphorylation of downstream signaling molecules Akt and Erk1/2 was observed upon transgene activation in both tumor tissue and RM11A cells [21,43]. *In vivo*, these cells were shown to form tumors upon injection into the mammary gland of syngeneic, wild type, FVB mice. Because of its inducible nature, our model can be used to mimic the effects of IGF-IR-directed therapies through the deactivation of the transgene, and therefore provides a unique opportunity to study the potential function of other known oncogenes during IGF-IR-mediated mammary tumorigenesis. As a number of IGF-IR inhibitory compounds are currently in clinical trials [23,44], this model can be used to predict how the inhibition of other proteins/pathways could be utilized to augment IGF-IR-directed therapeutics.

It has been observed that ErbB2 overexpression can alleviate the requirements of IGF and EGF for proliferation in a series of human normal and breast cancer cell lines [45]. However, most studies have focused on IGF signaling as a mechanism through which clinical cancers gain resistance to ErbB2-directed therapies. It remains unclear whether the reciprocal is also true; signaling through ErbB2 can potentially promote resistance to IGF-IR-targeted treatments. Also, with the prevalence of both oncogenes overexpressed in human breast cancer, it is important to investigate their interaction in mammary tumorigenesis to determine whether it would be useful to combine therapeutics directed at both ErbB2 and IGF-IR. Thus, our objective was to determine if ErbB2 overexpression can augment tumorigenesis and compensate for IGF-IR downregulation in RM11A cells, a model of IGF-IR-mediated mammary tumorigenesis. We accomplished this through overexpression of ErbB2 in the RM11A mammary cell line. We then examined how ErbB2 contributed to cell survival/growth *in vitro *and *in vivo*, cell signaling, primary tumorigenesis, recurrence in the absence of IGF-IR transgene expression and metastasis. In this study, it was determined that a modest increase in ErbB2 expression could accelerate primary tumor growth by enhancing proliferation immediately after cell colonization of the mammary gland. Overexpression of ErbB2 also impaired regression of tumors in the absence of IGF-IR transgene expression and facilitated metastasis.

## Methods

### ErbB2 overexpression construct

A wild type rat ErbB2 (*neu*) expression construct (described in [46]) was a generous gift from William Muller, McGill University, Montreal, QC. Briefly, this expression construct contained ErbB2 cDNA under the control of the Moloney murine leukemia virus promoter which was cloned into the *Eco*RI and *Hind*III sites of pEGFP-N1 (Clontech, Mountain View, CA). This ErbB2 expression plasmid was referred to as pEN1-ErbB2, while the empty vector was referred to as pEN1. Plasmid DNA was purified using a Qiagen mini-prep kit (Qiagen, Mississauga, ON, Canada) in accordance with the manufacturer's instructions.

### Western Blotting

Western blotting was performed as previously described [43]. Primary antibodies used were anti-neu (C-18) (Santa Cruz Biotechnologies, Santa Cruz, CA) and anti-C-ErbB2 (pTyr^1222^) (AnaSpec, Fremont, CA) both used at a concentration of 1:250, anti-phospho-Akt, anti-Akt, anti-phospho-Erk1/2 and anti-Erk1/2 (Cell Signal technology, Danvers, MA) were all used at a dilution of 1:1,000, as well as anti-IGF-IR (R&D Systems, Minneapolis, MN) used at a dilution of 1:1,000, and anti-β-actin used at 1:2,000. The secondary antibody used was anti-rabbit IgG (Cell Signal technology, Danvers, MA) and was used at a dilution of 1:2,000. Densitometry of the bands was quantified using a FluorChem 9900 imaging system and AlphaEaseFC software version 3.1.2 (Alpha Innotech, San Leandro, CA). Densitometry values were normalized to those of the loading control, β-actin, and these normalized numbers were expressed as values relative to the control.

### Cell line and culture conditions

RM11A cells, a cell line previously derived from a tumor from an MTB-IGF-IR mouse, were maintained in media as described previously [43]. Cells were continually cultured in media supplemented with 10 μg/mL of doxycycline (Sigma-Aldrich, St. Louis, MO) to maintain high levels of IGF-IR transgene expression. Transfection was performed with lipofectamine2000 transfection reagent (Invitrogen, Burlington, ON, Canada) in accordance with the manufacturer's protocol. For ErbB2 overexpression, RM11A cells were transfected with pEN1-ErbB2 (these cells were called RM11A+Dox/ErbB2); in addition, cells were transfected with the empty vector to serve as a control (these cells were called RM11A+Dox). To select clones with stable integration of the aforementioned plasmids, cell culture media was supplemented with G418 (Sigma-Aldrich, St. Louis, MO) at a final concentration of 750 μg/mL, a concentration previously validated to kill 100% of untransfected RM11A cells in 3-4 days. After approximately two weeks individual colonies were isolated and tested for increased ErbB2 expression by Western blotting and subsequently maintained with G418. Two control clones and two clones showing a consistent elevation of ErbB2 were selected and used for further experiments.

### MTT cell survival assays

One thousand RM11A+Dox or RM11A+Dox/ErbB2 cells/well were plated in triplicate in 96-well plates. Forty-eight hours after plating the cells were incubated with MTT at a final concentration of 5 mg/mL for 1 h at 37°C. Cells were then lysed and the absorbance value at 570 nm was determined. Results represent the average of seven replicates.

### H&E, Immunohistochemistry and immunofluorescence

H&E staining was performed as previously described [43]. To measure tumor area from H&E stained sections, slides were scanned using an Aperio Scanscope (Aperio Technologies, Vista, CA) digital slide scanner, and measurements were performed using ImageScope software v. 10.0.36.1805 (Aperio Technologies, Vista, CA).

Immunofluorescence was used to assess proliferation. Cells were plated on glass coverslips in 6-well plates at a density of 3 × 10^4 ^cells/well. Two days after plating, cells were fixed and stained as described previously [47]. The primary antibody anti-Ki67 (Abcam, Cambridge, MA) was used at a dilution of 1:200, while the fluorescent conjugated secondary antibody, anti-Rabbit IgG, was used at a dilution of 1:1,000. Images were captured using an Olympus BX61 fluorescent microscope (Center Valley, PA) and MetaMorph version 7.6.0.0 software (Molecular Devices, Downington, PA) at a magnification of 200×; positively stained nuclei were counted from 15-20 fields of view comprising approximately 400-1,000 cells and the proportion of cell nuclei staining positive for Ki67 was reported. These experiments were repeated in triplicate.

Immunohistochemistry was performed as previously described [47]. Anti-Ki67 (Abcam, Cambridge, MA) was used at a concentration of 1:100. Anti-ErbB2 (Santa Cruz Biotechnologies, Santa Cruz, CA) was used at a dilution of 1:100. Appropriate biotinylated secondary antibodies were used at a concentration of 1:100. For analysis of Ki67 staining, tumor images were collected at a magnification of 100× and the proportion of positively stained nuclei was determined for a minimum of 5 fields of view.

### Animal trials

All mice were housed and utilized following the guidelines established by the Animal Care Committee at the University of Guelph and the Canadian Council on Animal Care. Wild type FVB mice were purchased from Charles River (Wilmington, MA). At approximately 4 weeks of age, animals were anesthetized and both 4^th ^inguinal mammary glands were injected with 5 × 10^5 ^RM11A+Dox or RM11A+Dox/ErbB2 cells resuspended in 10 μL of PBS using a 25 μL Hamilton syringe as described in [43]. Mice were treated with doxycycline food pellets 2 g/kg (Bio-Serv, Frenchtown, NY) to maintain IGF-IR overexpression. After tumor cell injection, mice were either sacrificed after 4 or 14 days or when the tumors reached 15-17 mm in length (the maximum tumor length allowed by the Canadian Council for Animal Care is 17 mm). For the latter condition, mice were monitored for palpable tumors 2 times per week; tumor onset was recorded for both mammary glands and palpable tumors were measured (length and width) with digital calipers. Tumor volume was calculated using the equation: *volume = length × width*^*2*^/*2*. To track tumor growth, two methods were used. The first method was calculating specific growth rate (SGR), an established method for this measurement [48]. In addition, the slope of the line of log_10_(tumor volume) versus time(d) was used to calculate tumor doubling time for validation. For the tumor regression studies IGF-IR expression was downregulated when tumors reached 7-11 mm in length by removing doxycycline from the animals' diets. Subsequent regression and recurrence in the absence of IGF-IR transgene expression was monitored as above.

MTB-IGFIR double transgenic mice were used as previously described [1]. Lungs were collected when primary tumors reached a maximum of 17 mm in length. H&E was performed to locate microscopic lung lesions as described below.

### Evaluation of metastasis

Tissue comprising the entire lung from each mouse harboring a 15-17 mm length tumor was collected and processed as described above. Approximately 25 serial sections were taken from the middle of each lung. H&E was performed on sections from the beginning and second half of the series. Slides were evaluated for the presence of metastases using light microscopy by two individuals in a blinded manner.

### Statistics

Data values are presented as the mean ± SE. Significance and p-values were obtained using a Student's t-test. Differences in metastasis were assessed using a Fisher's exact test. Significance between tumor regression data was calculated using a chi-squared test.

## Results

### Stable overexpression of ErbB2

**Figure 1:**
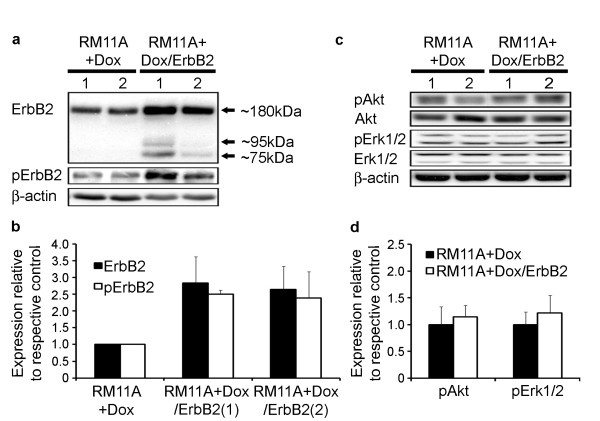
**ErbB2 overexpression in RM11A cells did not affect activation of Akt and Erk**. ErbB2 and phosphorylated ErbB2 overexpression were consistently observed by Western blotting in two independent stable clones transfected with the ErbB2 expression construct (RM11A+Dox/ErbB2) (a). ErbB2 expression was quantified after Western blotting and normalized to the loading control β-actin. Overexpression of both ErbB2 and pErbB2 was ~2-3 fold higher in both RM11A+Dox/ErbB2 clones compared to RM11A+Dox clones (b). Representative Western blots of pAkt and pErk (c). Average relative pAkt and pErk levels were determined to be unchanged in RM11A+Dox/ErbB2 cells compared to RM11A+Dox cells (d).

The ability of doxycycline to induce elevated IGF-IR levels in the RM11A cells as well as their *in vitro *and *in vivo *growth characteristics have previously been reported [43]. To examine how concomitant overexpression of IGF-IR and ErbB2 effects mammary tumorigenesis, ErbB2 was overexpressed in the previously characterized IGF-IR inducible RM11A cell line [43]. Two independent transfections of pEN1-ErbB2 (an ErbB2 overexpression construct) and pEN1 (the corresponding control plasmid) with subsequent culture in media containing G418 resulted in the stable selection of two pEN1-ErbB2 plasmid integrants and two pEN1 control stable transfectants. RM11A cells overexpressing IGF-IR but containing the control plasmid were designated RM11A+Dox while the RM11A cells co-overexpressing IGF-IR and ErbB2 were designated RM11A+Dox/ErbB2. Western blotting was used to confirm elevated expression of ErbB2 in RM11A cells transfected with the pEN1-ErbB2 plasmid (RM11A+Dox/ErbB2). Ultimately one RM11A clone from each selection procedure showing the highest and most consistent overexpression of ErbB2 (RM11A+Dox/ErbB2) was chosen for further studies. In addition, two empty vector stable integrants were generated in parallel to be used as controls (RM11A+Dox). Average overexpression of ErbB2 was approximately 2.5-3-fold compared to RM11A+Dox cells (Figure 1a, b). Interestingly, two additional bands were observed in both RM11A+Dox/ErbB2 clones at approximately 75 and 90 kDa that were not observed in RM11A+Dox cells (Figure 1a). In addition, Western blots revealed that phospho-ErbB2 levels were consistently increased in RM11A+Dox/ErbB2 cells compared to RM11A+Dox cells by the same proportion as ErbB2 was upregulated (Figure 1a, b). Western blotting of signaling molecules downstream of IGF-IR and ErbB2 revealed that RM11A+Dox/ErbB2 cells had similar levels of phosphorylated Akt and Erk1/2 as RM11A+Dox cells (Figure 1c, d). Therefore, ErbB2 expression was elevated in RM11A+Dox/ErbB2 cells and ErbB2 was functional as indicated by the increase in phosphorylated ErbB2.

### Overexpression of ErbB2 did not affect survival/proliferation in RM11A cells *in vitro*

We hypothesized that ErbB2 overexpression would enhance cell survival and proliferation. Survival of RM11A+Dox and RM11A+Dox/ErbB2 cells was assessed by MTT assays, while proliferation was quantified using Ki67 immunofluorescence. ErbB2 overexpression did not significantly affect proliferation or survival *in vitro *(data not shown). Therefore, elevated ErbB2 expression cannot further enhance survival or proliferation beyond that of the effect demonstrated by IGF-IR overexpression.

### ErbB2 enhances tumorigenesis *in vivo*

**Figure 2:**
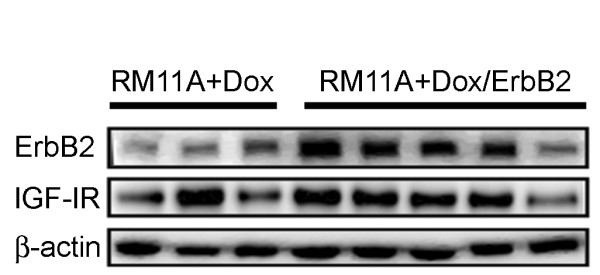
**RM11A+Dox/ErbB2 cells maintain ErbB2 and IGF-IR overexpression *in vivo***. Western blot analysis of IGF-IR and ErbB2 in RM11A+Dox cells expressing high levels of IGF-IR and RM11A+Dox/ErbB2 cells expressing high levels of both IGF-IR and ErbB2 following *in vivo *tumor growth. β-actin served as a loading control. Tissue was collected when tumors reached 17 mm in length.

To examine the effects of ErbB2 in a model of IGF-IR driven mammary tumorigenesis *in vivo*, RM11A+Dox and RM11A+Dox/ErbB2 cells were injected into the mammary fat pad of wild type syngeneic FVB mice and tumor onset and growth rates were evaluated. RM11A+Dox/ErbB2 cells produced palpable mammary tumors approximately 22 days post injection. This latency was significantly shorter than the time required for the RM11A+Dox cells to form palpable tumors (48 days) (Table 1). Western blotting confirmed that elevated ErbB2 expression in the RM11A+Dox/ErbB2 cells was maintained *in vivo*; IGF-IR overexpression was also maintained in tumors derived from both RM11A+Dox and RM11A+Dox/ErbB2 cells (Figure 2). ErbB2 overexpression was also capable of increasing the percentage of mammary glands that developed tumors (Table 1); RM11A+Dox/ErbB2 cells were more effective at producing tumors than RM11A+Dox cells. This data suggests that ErbB2 can augment IGF-IR mediated mammary tumorigenesis *in vivo *by both increasing tumor incidence and decreasing tumor lactency.

**Table 1:** Summary of tumor development in FVB mice injected with RM11A stable transfectants

	RM11A+Dox	RM11A+Dox/ErbB2
Number of mice injected	38	31

Number of palpable tumors	29	42

Number of tumors per mouse	0.76	1.35

Average tumor latency (d)	47.9	22.1^a^

^a ^Significantly lower than RM11A-+Dox (p < 0.001)

**Figure 3:**
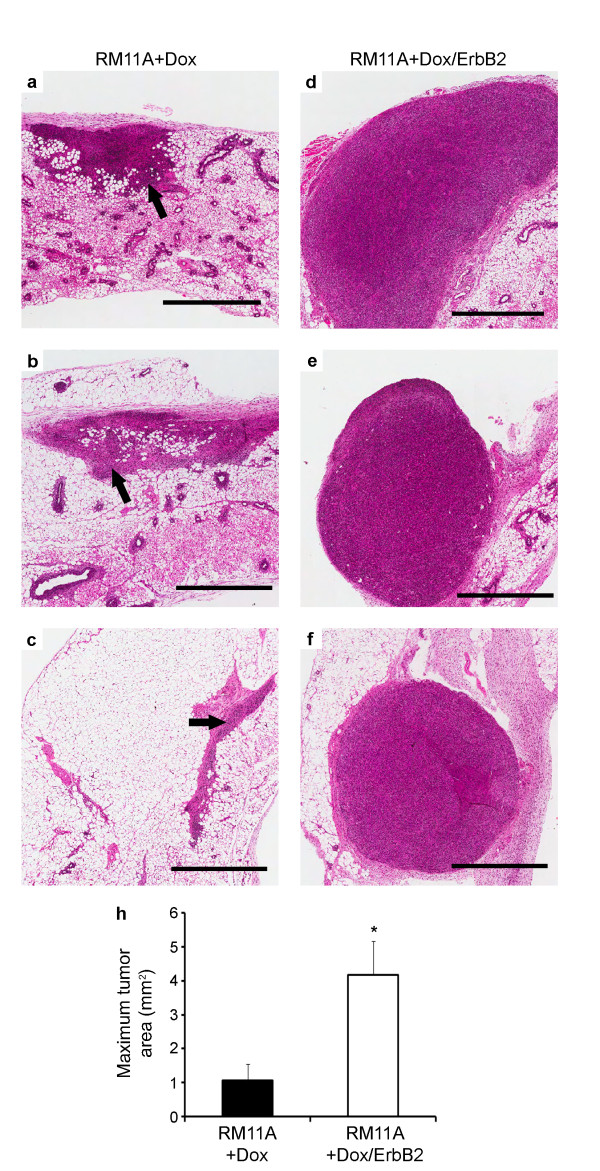
**ErbB2 overexpression enhanced tumor size with IGF-IR induction after 14 days**. H&E stained mammary sections 14 days after injection of RM11A+Dox injected mammary glands (a, b, c) and RM11A+Dox/ErbB2 injected mammary glands (d, e, f) are shown. Scale bars: 1 mm for (a-f). Quantification of tumor area is presented in (h). Asterisk = p < 0.05.

To corroborate the tumor onset data, a subset of mice receiving doxycycline were sacrificed 14 days post tumor cell injection and the mammary glands were histologically assessed using hematoxylin and eosin stained sections. As seen in Figure 3a-f, tumors from RM11A+Dox/ErbB2 cells (Figure 3d-f) were larger than tumors from RM11A+Dox cells (Figure 3a-c). The average tumor area was approximately 4-fold higher in the RM11A+Dox/ErbB2 tumors compared to RM11A+Dox tumors at this time point (Figure 3h).

### ErbB2 enhances proliferation in RM11A cells *in vivo *only after initial mammary gland colonization

**Figure 4:**
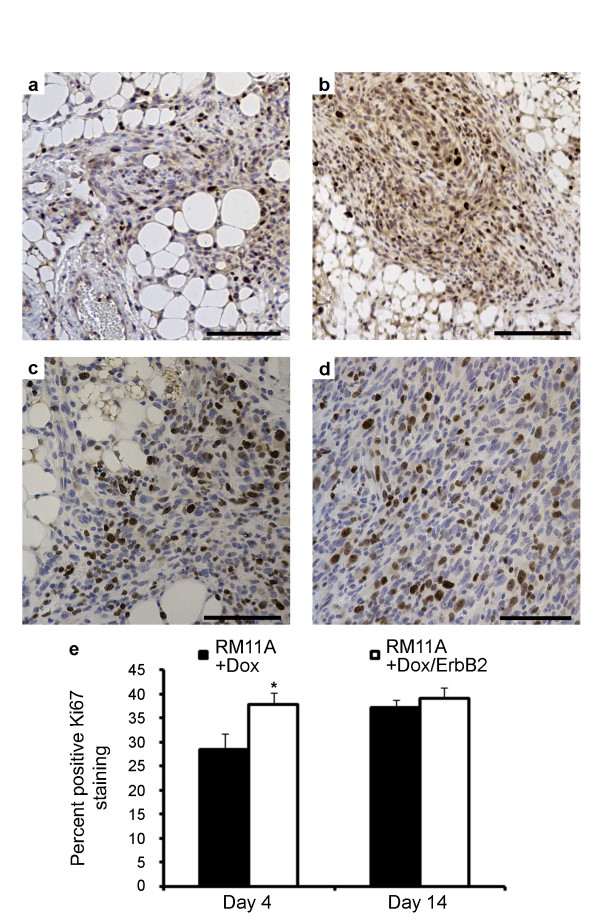
**ErbB2 overexpression enhanced proliferation *in vivo *shortly after injection**. Immunohistochemistry for Ki67 in mammary glands injected with RM11A+Dox (a, c) and RM11A+Dox/ErbB2 (b, d) cells 4 (a, b) or 14 (c, d) days after cell injection into the mammary fat pad. Quantification of Ki67 staining revealed a significant increase in proliferation at day 4 but not day 14 in the RM11A+Dox/ErbB2 tumors compared to RM11A+Dox tumors (e). Scale bars: 100 μm.

To test our hypothesis that ErbB2 overexpression enhanced initial growth of tumor cells, proliferation was determined using Ki67 immunohistochemistry at four or 14 days post tumor cell injection. As shown in Figure 4, proliferation was higher in RM11A+Dox/ErbB2 cells (Figure 4b) compared to RM11A+Dox cells (Figure 4a) at 4 days post cell injection. Upon quantitative analysis, a statistically significant 1.3-fold increase in Ki67 positivity was observed in the RM11A+Dox/ErbB2 tumor cells compared to RM11A+Dox tumor cells (Figure 4e). However, there were no significant difference in Ki67 staining between RM11A+Dox tumor cells (Figure 4c) and RM11A+Dox/ErbB2 tumor cells 14 d post injection (Figure 4d), suggesting that ErbB2 increases tumor growth rate during the first few days of tumor development but not once tumors have been established. In addition, no correlation was observed between tumor size and percent Ki67 staining (data not shown), indicating that proliferation was not dependent on tumor size.

Two methods were used to further quantify tumor growth rate after the formation of palpable nodules; SGR and tumor doubling time. As seen in Table 2, there was no significant difference in average SGR values in tumors induced by RM11A+Dox cells compared to RM11A+Dox/ErbB2 cells. Similarly, doubling time was not accelerated by ErbB2 addition. These results strongly indicate that ErbB2 overexpression does not enhance tumor growth rate once the tumors become large enough to be palpated.

**Table 2:** Summary of tumor growth rate in FVB mice injected with RM11A-ErbB2 stable clones

	RM11A+Dox	RM11A+Dox/ErbB2
Specific growth rate (%/d)	17.9 ± 2.0	15.8 ± 1.4

Doubling time (d)	4.4 ±0.2	5.4 ± 0.4^a^

^a ^Significantly higher than RM11A+Dox (p < 0.05)

### ErbB2 overexpression impairs regression and enhances metastasis following IGF-IR downregulation

Previously it has been observed that most tumors formed after injection of RM11A+Dox cells into the mammary gland regress following IGF-IR downregulation, with most of these tumors recurring independent of IGF-IR transgene expression [49]. To examine the effect of ErbB2 overexpression on tumor regression in our model, RM11A+Dox and RM11A+Dox/ErbB2 cells were injected into the mammary gland of wild type mice. Once tumors reached 7-11 mm in length (approximately 150-450 mm^3 ^in volume) IGF-IR transgene expression was suppressed by switching the animals from a doxycycline diet to a normal diet (animals remained on a normal diet for the remainder of the study). Tumor length prior to IGF-IR downregulation did not significantly vary between the RM11A+Dox and RM11A+Dox/ErbB2 groups. As shown in Table 3, the presence of ErbB2 impaired tumor regression following IGF-IR transgene downregulation (doxycycline withdrawal). RM11A cells with elevated ErbB2 levels failed to completely regress and half of the tumors failed to even partially regress. In contrast, 27% of RM11A+Dox tumors completely regressed following IGF-IR transgene downregulation and only 9% of the tumors failed to at least partially regress (Table 3). Using a chi-square test, the difference in tumor regression characteristics was determined to be statistically significant (p < 0.05).

**Table 3:** Summary of tumor regression after IGF-IR downreguation (% reduction in volume)

	Number of tumors with full regression (100%)	Number of tumors with partial regression (10-99.9%)	Number of tumors with no/minimal regression (0-10%)
RM11A+Dox	3/11 (27.2%)	7/11 (63.6%)	1/11 (9.1%)

RM11A+Dox/ErbB2	0/14 (0.0%)	7/14 (50.0%)	7/14 (50.0%)

Significantly different between the two conditions tested (p < 0.05)

**Figure 5:**
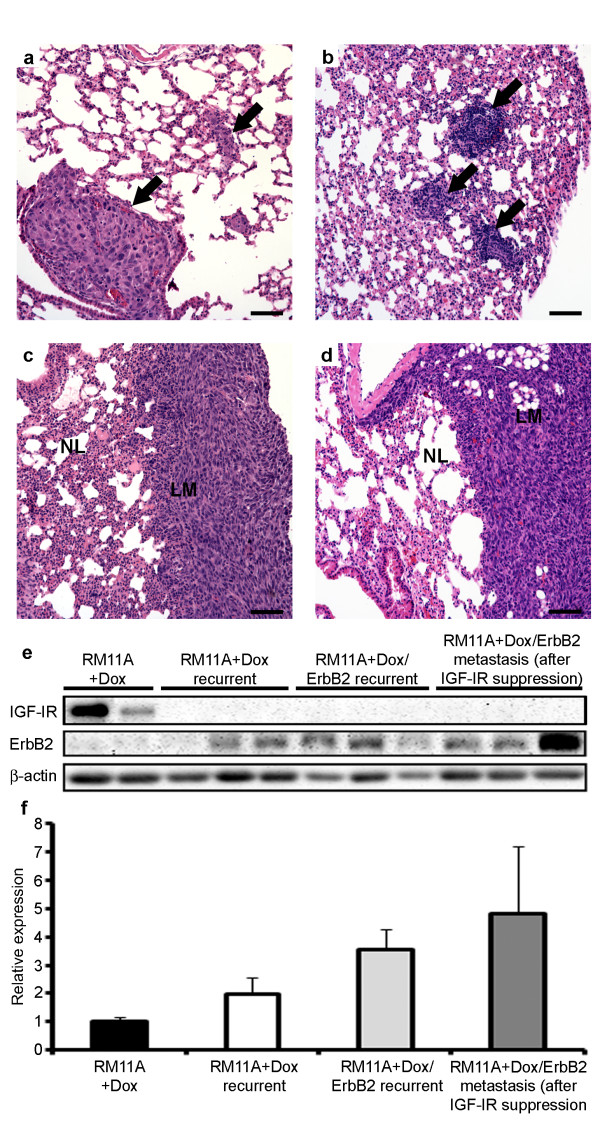
**Metastasis was observed after injection of RM11A variants**. H&E stained sections of lung tissue with metastasis following intra-mammary injection and subsequent mammary tumor development of RM11A+Dox (b) or RM11A+Dox/ErbB2 injected mice (a) are shown (arrows). Typically these lesions were relatively small. Metastasis was also observed to occur following IGF-IR downregulation in RM11A+Dox/ErbB2-injected animals and these lesions were usually larger than aforementioned lesions (c, d); (LM = lung metastasis; NL = normal lung). Scale bars: 100 μm. Western blotting was performed on protein lysates from RM11A+Dox and RM11A+Dox/ErbB2 doxycycline-independent recurrent tumors and metastases to confirm IGF-IR and ErbB2 levels (e). Relative quantification is shown in (f).

To determine whether ErbB2 overexpression altered the metastatic capacity of RM11A cells, lung tissue from mice harboring primary tumors (15-17 mm in length) or tumors that recurred following IGF-IR transgene downregulation (15-17 mm in length) was analyzed. As shown in Table 4, the incidence of microscopic lung metastasis from primary mammary tumors derived from RM11A+Dox cells was relatively infrequent with only 3 of 21 mice developing lung metastases. Overexpression of ErbB2 in the presence of high IGF-IR expression did not enhance metastasis to the lung as only 2 of 19 mice with primary tumors derived from RM11A+Dox/ErbB2 cells developed lung metastases. Representative images of metastasis resulting from the injection of RM11A+Dox and RM11A+Dox/ErbB2 are shown in Figure 5a and 5b respectively.

**Table 4:** Summary of metastasis in FVB mice injected with RM11A-ErbB2 stable transfectants

	RM11A+Dox	RM11A+Dox/ErbB2	RM11A+Dox after IGF-IR suppression	RM11A+Dox/-ErbB2 after IGF-IR suppression
Number of mice	21	19	12	13

Number with microscopic lung metastasis	3	2	0	6

Percent with metastasis	14.3	10.5	0.0	46.2^a^

Number of mice with macroscopic lung metastasis	0	0	0	3

Number of mice with macroscopic liver metastasis	0	0	0	1

^a ^Significantly different than RM11A+Dox after IGF-IR suppression (p < 0.05)

Lung metastasis was also examined in mice harboring RM11A+Dox and RM11A+Dox/ErbB2 tumors that grew following IGF-IR transgene downregulation. Six of 13 tumors expressing high levels of ErbB2 metastasized to the lung while none of the tumors with basal ErbB2 expression metastasized to the lung (Figure 5c, d). In addition, these lung metastases were macroscopic and liver metastases were also observed (liver metastases have never been observed previously in RM11A cells). Confirmation of IGF-IR downregulation in doxycycline-independent recurrent tumors and metastases as well as upregulation of ErbB2 in RM11A+Dox/ErbB2 doxycycline-independent tumors and metastases is shown in Figure 5e, f. Therefore, tumors initially expressing high levels of both IGF-IR and ErbB2 (RM11A+Dox/ErbB2) are metastatic and this metastatic potential appears to increase following IGF-IR downregulation.

### ErbB2 is overexpressed in metastatic lesions from MTB-IGFIR transgenic mice

**Figure 6:**
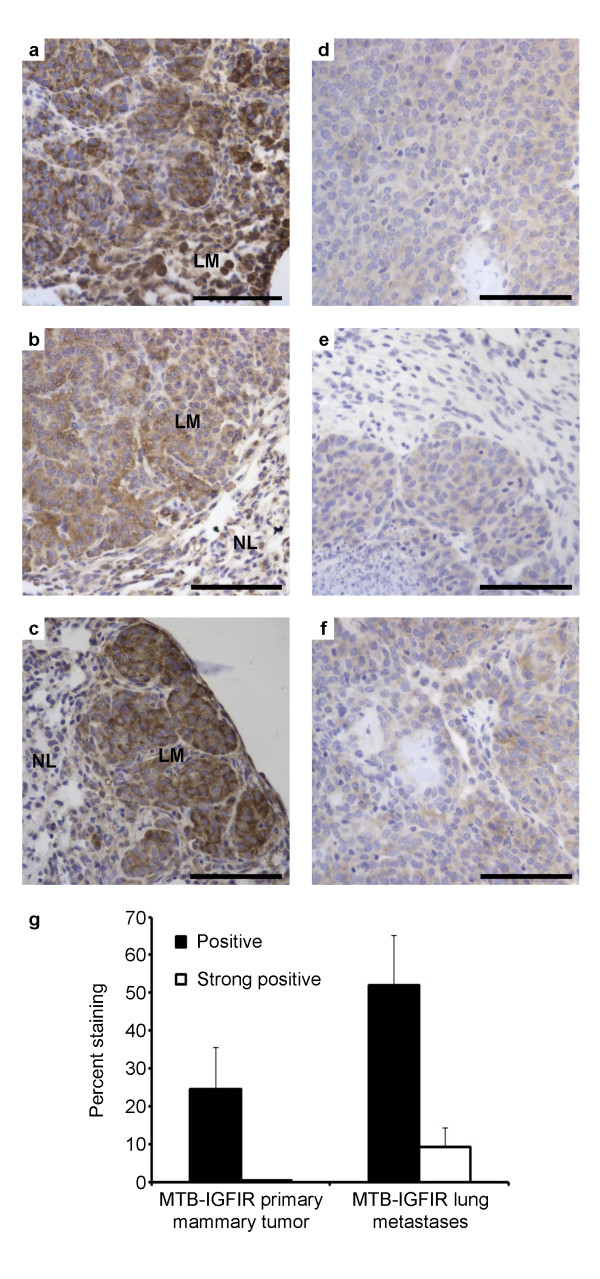
**Metastatic lesions from MTB-IGFIR transgenic mice display elevated ErbB2 expression**. Representative ErbB2 staining in lung sections (a, b, c) and primary tumors (d, e, f); LM denotes the metastatic lung lesion, while NL refers to surrounding normal lung tissue. Quantitative analysis revealed that an approximate 2-fold higher level of positivity (p = 0.15) and a 30-fold increase in strong positive labeled cells (p = 0.16) in metastases compared to primary tumors (g).

**Figure 7:**
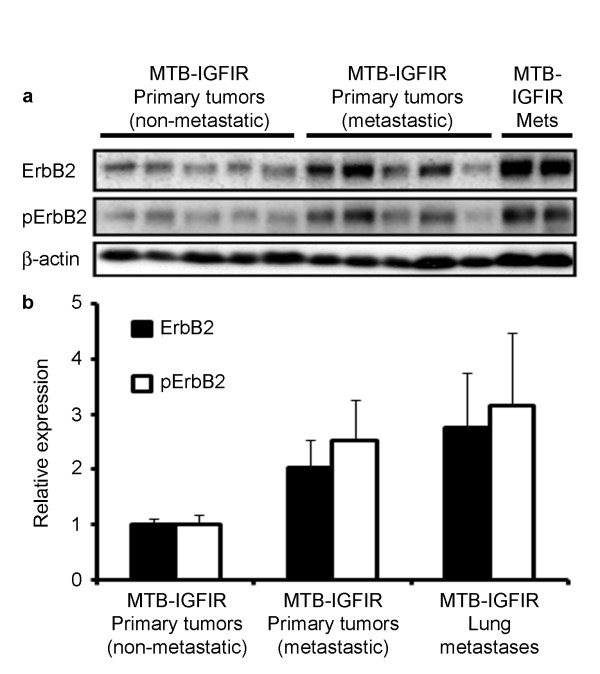
**ErbB2 was upregulated in metastatic tumors**. Western blotting for ErbB2 and pErbB2 in primary mammary tumors from MTB-IGFIR mice that did not metastasize, primary mammary tumors that did metastasize and isolated lung metastases (a). Densitometry revealed that ErbB2 and pErbB2 levels were approximately 2-fold higher in tumors from mice with metastases (p = 0.10 for both ErbB2 and pErbB2) and approximately 3-fold higher in metastatic lesions (p = 0.09 for ErbB2 and p = 0.10 for pErbB2) compared to primary mammary tumors that did not metastasize (b).

Metastasis to the lung has been observed in approximately 40% of MTB-IGFIR transgenic mice harboring tumors 15-17 mm in length and these metastases range in size from microscopic lesions of approximately 50-100 μm in length to macroscopic tumors approximately 6-8 mm in length (unpublished observations). To determine whether ErbB2 is involved in metastasis of mammary tumors produced by MTB-IGFIR transgenic mice, immunohistochemistry for ErbB2 was performed on the aforementioned lung tissue. While a relatively high level of variability was observed in both primary tumors and microscopic lung metastases, there was a tendency for lung lesions to stain more intensely for ErbB2 than primary tumors (Figure 6a-c and 6d-f respectively). Through quantification of staining, this was verified as there was an approximate 2-fold increase in positive staining in metastases (Figure 6g). Some of the larger lung lesions were observed to have especially intense ErbB2 staining (examples shown include Figure 6a, c); this translated to a 30-fold increase in strong positive staining in metastatic lesions compared to primary mammary tumors (Figure 6g). In metastatic lesions from MTB-IGFIR mice, the levels of ErbB2 and the activation status of ErbB2 were confirmed using western blots. Isolated lung metastases displayed very high levels of ErbB2 and phosphorylated ErbB2 (Figure 7a, b). Interestingly, primary mammary tumors from mice with lung metastases also displayed high levels of ErbB2 and phosphorylated ErbB2 compared to primary mammary tumors from mice with no evidence of lung metastases (Figure 7a, b). Taken together these data suggest that metastatic tumor cells express elevated levels of ErbB2.

## Discussion

While the contribution of the IGF-IR to ErbB2 signaling and resistance to ErbB2-directed therapies in breast cancer has been studied in several systems, the reciprocal interaction remains almost completely unknown. To study the potential role of ErbB2 during IGF-IR-mediated mammary tumorigenesis we utilized our model of inducible IGF-IR overexpression. The importance of the IGF-axis in proliferation and transformation of a vast number of cells including human mammary epithelial cells is well documented. Our laboratory has shown that IGF-IR overexpression alone is capable of mediating an extremely rapid transformation of mouse mammary epithelial cells [21]. Most of these tumors remained dependent on transgene expression as IGF-IR transgene downregulation resulted in tumor regression in a majority of these tumors. A small subset of tumors was capable of resuming growth in the absence of IGF-IR transgene expression [49]. Similarly, RM11A cells grown in the mammary fat pad of syngeneic wild type mice developed tumors more rapidly when IGF-IR transgene expression was induced and IGF-IR transgene downregulation resulted in the regression of most of the tumors. Unlike the transgenic mammary tumors, most of the RM11A induced tumors eventually resumed growth following IGF-IR transgene downregulation [43]. Therefore, while IGF-IR-induction remains very important for mammary tumorigenesis in our model, clearly alterations in the expression of other genes/proteins can be acquired during tumorigenesis, which allow tumors to grow independent of the IGF-IR transgene. Given the prominent role of ErbB2 in breast cancer and the previously discussed interactions with the IGF-IR, this oncogene was an attractive candidate.

Selection of stable transfectants yielded RM11A cells with approximately 3-fold higher expression of ErbB2 (RM11A+Dox/ErbB2) than control RM11A cells (RM11A+Dox). Phosphorylated ErbB2 was also elevated approximately 3-fold in the ErbB2 overexpressing cells thus indicating the receptor was active. This overexpression was monitored and consistently maintained throughout the duration of the study (representative western blot is shown in Figure 1). It is well understood that breast cancer tissue and cell lines can contain upwards of 50-100-fold higher levels of ErbB2 protein compared to normal tissue and these levels are typically associated with gene amplification and poor prognosis [50,51]. While a modest overexpression was achieved in our cell line, two points should be taken into consideration; first, RM11A cells intrinsically contain high levels of ErbB2. In addition, recent studies have shown a correlation between low-level ErbB2 expression (comparable to normal mammary tissue) and an overall unfavorable disease outcome [52,53].

To control for phenotypes caused by integration of the plasmid, we utilized two separate clones for both control and ErbB2 overexpressing stable integrants. Interestingly, the ErbB2 antibody detected two additional bands only in clones with pEN1-ErbB2 integration (and not with the control plasmid). These bands were determined to be approximately 95 and 70 kDa. A 95 kDa truncated N-terminal product of Her2, known as p95Her-2, has been previously described [54]. This degradation product arises from proteolytic cleavage and subsequent release of the extracellular domain [55,56]. While the function of this protein is largely unknown, it has been shown to contribute to herceptin resistance [57]. In addition, it was observed that higher levels of this product correlated with shorter disease free survival and increased lymph node metastasis [58-60]. Thus, the presence of this ErbB2 degradation product appears to contribute to tumorigenesis. In our cell line, the presence of such a product would indicate that stable integration of this expression vector has yielded a threshold level of ErbB2 expression required for significant production of this cleavage product.

Downstream signaling pathways were studied to determine those potentially augmented by ErbB2 overexpression. The levels of phosphorylated Akt and Erk1/2 were similar in RM11A+Dox cells and RM11A+Dox/ErbB2 cells suggesting that upregulation of ErbB2 was incapable of further activating PI-3K or MAPK pathways. Given the magnitude of IGF-IR overexpression and the fact that both of these pathways are known to be activated by this receptor this observation is not surprising; it is anticipated that the high level of IGF-IR expression has already maximized signaling though the PI-3K and MAPK pathways [43].

*In vivo*, it was observed that ErbB2 conferred a more rapid tumor onset and tumor incidence was also elevated as indicated by the number of mammary glands injected that actually developed tumors. To verify that ErbB2 shortened tumor latency, mammary glands were collected 14 d post-injection. Average tumor size at this time point was 4-fold greater in the RM11A+Dox/ErbB2 cells compared to RM11A+Dox cells. We then explored possible mechanisms through which ErbB2 augmented tumor growth. First we looked at survival and proliferation *in vitro*. Only a small, insignificant increase in survival was observed in RM11A+Dox/ErbB2 cells were compared to RM11+Dox cells and thus it was concluded that ErbB2 overexpression had a negligible effect on RM11A cell survival *in vitro*. Despite a minimal effect *in vitro*, overexpression of ErbB2 had a marked effect on tumorigenesis *in vivo*. Using Ki67 staining to examine proliferation our data suggested that proliferation is only significantly affected by ErbB2 overexpression shortly after tumor cells colonize the mammary tissue (4 d post injection but not 14 d post injection). The lack of difference in proliferation in established tumors was corroborated by evaluating tumor growth rates using two independent methods. For the first method log(tumor volume) was plotted against time and from the resulting slope of the line tumor doubling time was calculated. The second technique, specific growth rate has been mathematically determined to be an accurate means of quantifying tumor growth rate and is less susceptible to negligible or negative changes in volume from one measurement to the next [61]. In this experiment, tumor volumes were sometimes observed to decrease before ultimately increasing again and thus this model of growth rate was deemed appropriate. Both methods yielded similar results and showed that ErbB2 overexpression did not enhance tumor growth rate. Therefore, it was concluded that in the presence of IGF-IR overexpression, ErbB2 facilitates proliferation during initial tumor establishment but has no effect on tumor growth/proliferation after this point.

Tumor regression following IGF-IR transgene downregulation was studied to model the effects of ErbB2 overexpression during the use IGF-IR-directed therapeutics. Here it was observed that ErbB2 overexpression impaired tumor regression following IGF-IR downregulation thus suggesting that ErbB2 could potentially facilitate resistance to IGF-IR-directed therapies. These results are of obvious clinical importance as ErbB2 status may become an important predictor of response to IGF-IR-directed therapies. In addition, subsequent mutations enhancing ErbB2 expression may render tumors unresponsive to these therapies. It is becoming clear that IGF-IR can mediate resistance to ErbB2-targeting treatments [33]; therefore, it stands to reason that the reciprocal interaction may also be important.

Metastasis was also studied for multiple reasons; first, ErbB2 expression is well known to correlate with distant metastasis in human clinical breast cancer [62]. Also, overexpression of this oncogene has been shown to increase the metastatic potential of human breast cancer cell lines [63]. Lastly, in transgenic models of ErbB2 overexpression, metastasis to the lung is a common occurrence [2]. Contrary to our expectations, ErbB2 overexpression did not facilitate an increase in lung metastasis. This could be due to the rapid establishment, onset and subsequent growth rate in primary tumors formed with both IGF-IR and ErbB2 overexpression; for this condition time elapsed between tumor onsets and euthanizing the animals was only 31 days on average and sometimes as short as 15 days, while palpable tumor onset occurred as early as 7 days post injection.

ErbB2 overexpression did however facilitate metastasis following IGF-IR downregulation. It is possible that through the delayed process of partial regression and subsequent resumption of growth, metastasic lesions have time to grow to a size where they are detectable histologically. Furthermore, upregulation of ErbB2 was observed in metastatic primary tumors as well as many metastatic lesions from MTB-IGFIR mice compared to non-metastatic primary tumors. Based on the fact that metastasis is only observed in 40% of all MTB-IGFIR animals, it is apparent that other alterations must occur to confer metastatic competency. Our results suggest that upregulation of ErbB2 is one such mechanism through which tumor cells gain this capacity. These observations suggest that ErbB2 can compensate for the loss of IGF-IR signaling during mammary tumorigenesis and further supports a potential advantage in combining ErbB2 and IGF-IR-directed therapies.

In conclusion, this study describes experiments providing information regarding the interaction between two potent oncogenes in mammary tumorigenesis. It has been previously postulated that targeting multiple signaling pathways such as IGF-IR and ErbB2 may be beneficial to the treatment of breast cancer [35]. Based on our results, it is suggested that ErbB2 can augment IGF-IR-dependent mammary tumorigenesis by enhancing initial colonization and proliferation as well as following IGF-IR suppression.

## Competing interests

The authors declare that they have no competing interests.

## Authors' contributions

CC participated in design and coordination of the study as well as all of the experiments described in this study and drafting of the manuscript. JP participated in design of the study. RM coordinated the study and contributed to drafting of the manuscript. All authors have read and approved the final manuscript.

## References

[B1] Jones RA Moorehead RA The impact of transgenic IGF-IR overexpression on mammary development and tumorigenesis J Mammary Gland Biol Neoplasia 2008 13 407 413 10.1007/s10911-008-9097-1 19002570

[B2] Ursini-Siegel J Schade B Cardiff RD Muller WJ Insights from transgenic mouse models of ERBB2-induced breast cancer Nat Rev Cancer 2007 7 389 397 10.1038/nrc2127 17446858

[B3] Baserga R Hongo A Rubini M Prisco M Valentinis B The IGF-I receptor in cell growth, transformation and apoptosis Biochim Biophys Acta 1997 1332 F105 F126 9196021 10.1016/s0304-419x(97)00007-3

[B4] O'Connor R Regulation of IGF-I receptor signaling in tumor cells Horm Metab Res 2003 35 771 777 10.1055/s-2004-814166 14710357

[B5] Coppola D Ferber A Miura M Sell C D'Ambrosio C Rubin R A functional insulin-like growth factor I receptor is required for the mitogenic and transforming activities of the epidermal growth factor receptor Mol Cell Biol 1994 14 4588 4595 8007963 10.1128/mcb.14.7.4588-4595.1994 PMC358831

[B6] DeAngelis T Ferber A Baserga R Insulin-like growth factor I receptor is required for the mitogenic and transforming activities of the platelet-derived growth factor receptor J Cell Physiol 1995 164 214 221 10.1002/jcp.1041640126 7790393

[B7] Kaleko M Rutter WJ Miller AD Overexpression of the human insulinlike growth factor I receptor promotes ligand-dependent neoplastic transformation Mol Cell Biol 1990 10 464 473 2153917 10.1128/mcb.10.2.464-473.1990 PMC360815

[B8] Sell C Dumenil G Deveaud C Miura M Coppola D DeAngelis T Effect of a null mutation of the insulin-like growth factor I receptor gene on growth and transformation of mouse embryo fibroblasts Mol Cell Biol 1994 14 3604 3612 8196606 10.1128/mcb.14.6.3604-3612.1994 PMC358728

[B9] Happerfield LC Miles DW Barnes DM Thomsen LL Smith P Hanby A The localization of the insulin-like growth factor receptor 1 (IGFR-1) in benign and malignant breast tissue J Pathol 1997 183 412 417 10.1002/(SICI)1096-9896(199712)183:4<412::AID-PATH944>3.0.CO;2-4 9496257

[B10] Papa V Gliozzo B Clark GM McGuire WL Moore D Fujita-Yamaguchi Y Insulin-like growth factor-I receptors are overexpressed and predict a low risk in human breast cancer Cancer Res 1993 53 3736 3740 8339284

[B11] Resnik JL Reichart DB Huey K Webster NJ Seely BL Elevated insulin-like growth factor I receptor autophosphorylation and kinase activity in human breast cancer Cancer Res 1998 58 1159 1164 9515800

[B12] Lee AV Hilsenbeck SG Yee D IGF system components as prognostic markers in breast cancer Breast Cancer Res Treat 1998 47 295 302 10.1023/A:1005915420341 9516083

[B13] Law JH Habibi G Hu K Masoudi H Wang MY Stratford AL Phosphorylated insulin-like growth factor-i/insulin receptor is present in all breast cancer subtypes and is related to poor survival Cancer Res 2008 68 10238 10246 10.1158/0008-5472.CAN-08-2755 19074892

[B14] Schnarr B Strunz K Ohsam J Benner A Wacker J Mayer D Down-regulation of insulin-like growth factor-I receptor and insulin receptor substrate-1 expression in advanced human breast cancer Int J Cancer 2000 89 506 513 10.1002/1097-0215(20001120)89:6<506::AID-IJC7>3.0.CO;2-F 11102895

[B15] Arteaga CL Kitten LJ Coronado EB Jacobs S Kull FC Jr Allred DC Blockade of the type I somatomedin receptor inhibits growth of human breast cancer cells in athymic mice J Clin Invest 1989 84 1418 1423 10.1172/JCI114315 2553774 PMC304004

[B16] Burtrum D Zhu Z Lu D Anderson DM Prewett M Pereira DS A fully human monoclonal antibody to the insulin-like growth factor I receptor blocks ligand-dependent signaling and inhibits human tumor growth in vivo Cancer Res 2003 63 8912 8921 14695208

[B17] Chernicky CL Yi L Tan H Gan SU Ilan J Treatment of human breast cancer cells with antisense RNA to the type I insulin-like growth factor receptor inhibits cell growth, suppresses tumorigenesis, alters the metastatic potential, and prolongs survival in vivo Cancer Gene Ther 2000 7 384 395 10.1038/sj.cgt.7700126 10766344

[B18] Chernicky CL Tan H Yi L Loret dM Jr Ilan J Treatment of murine breast cancer cells with antisense RNA to the type I insulin-like growth factor receptor decreases the level of plasminogen activator transcripts, inhibits cell growth in vitro, and reduces tumorigenesis in vivo Mol Pathol 2002 55 102 109 10.1136/mp.55.2.102 11950959 PMC1187158

[B19] Salatino M Schillaci R Proietti CJ Carnevale R Frahm I Molinolo AA Inhibition of in vivo breast cancer growth by antisense oligodeoxynucleotides to type I insulin-like growth factor receptor mRNA involves inactivation of ErbBs, PI-3K/Akt and p42/p44 MAPK signaling pathways but not modulation of progesterone receptor activity Oncogene 2004 23 5161 5174 10.1038/sj.onc.1207659 15122317

[B20] Carboni JM Lee AV Hadsell DL Rowley BR Lee FY Bol DK Tumor development by transgenic expression of a constitutively active insulin-like growth factor I receptor Cancer Res 2005 65 3781 3787 10.1158/0008-5472.CAN-04-4602 15867374

[B21] Jones RA Campbell CI Gunther EJ Chodosh LA Petrik JJ Khokha R Transgenic overexpression of IGF-IR disrupts mammary ductal morphogenesis and induces tumor formation Oncogene 2007 26 1636 1644 10.1038/sj.onc.1209955 16953219

[B22] Haluska P Carboni JM TenEyck C Attar RM Hou X Yu C HER receptor signaling confers resistance to the insulin-like growth factor-I receptor inhibitor, BMS-536924 Mol Cancer Ther 2008 7 2589 2598 10.1158/1535-7163.MCT-08-0493 18765823 PMC2614316

[B23] Weroha SJ Haluska P IGF-1 receptor inhibitors in clinical trials--early lessons J Mammary Gland Biol Neoplasia 2008 13 471 483 10.1007/s10911-008-9104-6 19023648 PMC2728362

[B24] Penuel E Akita RW Sliwkowski MX Identification of a region within the ErbB2/HER2 intracellular domain that is necessary for ligand-independent association J Biol Chem 2002 277 28468 28473 10.1074/jbc.M202510200 12000754

[B25] Tzahar E Waterman H Chen X Levkowitz G Karunagaran D Lavi S A hierarchical network of interreceptor interactions determines signal transduction by Neu differentiation factor/neuregulin and epidermal growth factor Mol Cell Biol 1996 16 5276 5287 8816440 10.1128/mcb.16.10.5276 PMC231527

[B26] Pinkas-Kramarski R Soussan L Waterman H Levkowitz G Alroy I Klapper L Diversification of Neu differentiation factor and epidermal growth factor signaling by combinatorial receptor interactions EMBO J 1996 15 2452 2467 8665853 PMC450177

[B27] Yarden Y Sliwkowski MX Untangling the ErbB signalling network Nat Rev Mol Cell Biol 2001 2 127 137 10.1038/35052073 11252954

[B28] Slamon DJ Clark GM Wong SG Levin WJ Ullrich A McGuire WL Human breast cancer: correlation of relapse and survival with amplification of the HER-2/neu oncogene Science 1987 235 177 182 10.1126/science.3798106 3798106

[B29] Brandt R Wong AM Hynes NE Mammary glands reconstituted with Neu/ErbB2 transformed HC11 cells provide a novel orthotopic tumor model for testing anti-cancer agents Oncogene 2001 20 5459 5465 10.1038/sj.onc.1204709 11571643

[B30] Di Fiore PP Pierce JH Kraus MH Segatto O King CR Aaronson SA erbB-2 is a potent oncogene when overexpressed in NIH/3T3 cells Science 1987 237 178 182 10.1126/science.2885917 2885917

[B31] Hynes NE Lane HA ERBB receptors and cancer: the complexity of targeted inhibitors Nat Rev Cancer 2005 5 341 354 10.1038/nrc1609 15864276

[B32] Chakraborty AK Liang K Digiovanna MP Co-targeting insulin-like growth factor I receptor and HER2: dramatic effects of HER2 inhibitors on nonoverexpressing breast cancer Cancer Res 2008 68 1538 1545 10.1158/0008-5472.CAN-07-5935 18316619

[B33] Nahta R Yu D Hung MC Hortobagyi GN Esteva FJ Mechanisms of disease: understanding resistance to HER2-targeted therapy in human breast cancer Nat Clin Pract Oncol 2006 3 269 280 10.1038/ncponc0509 16683005

[B34] Balana ME Labriola L Salatino M Movsichoff F Peters G Charreau EH Activation of ErbB-2 via a hierarchical interaction between ErbB-2 and type I insulin-like growth factor receptor in mammary tumor cells Oncogene 2001 20 34 47 10.1038/sj.onc.1204050 11244498

[B35] Lu Y Zi X Zhao Y Mascarenhas D Pollak M Insulin-like growth factor-I receptor signaling and resistance to trastuzumab (Herceptin) J Natl Cancer Inst 2001 93 1852 1857 10.1093/jnci/93.24.1852 11752009

[B36] Nahta R Yu D Hung MC Hortobagyi GN Esteva FJ Mechanisms of disease: understanding resistance to HER2-targeted therapy in human breast cancer Nat Clin Pract Oncol 2006 3 269 280 10.1038/ncponc0509 16683005

[B37] Jerome L Alami N Belanger S Page V Yu Q Paterson J Recombinant human insulin-like growth factor binding protein 3 inhibits growth of human epidermal growth factor receptor-2-overexpressing breast tumors and potentiates herceptin activity in vivo Cancer Res 2006 66 7245 7252 10.1158/0008-5472.CAN-05-3555 16849573

[B38] Nahta R Yuan LX Zhang B Kobayashi R Esteva FJ Insulin-like growth factor-I receptor/human epidermal growth factor receptor 2 heterodimerization contributes to trastuzumab resistance of breast cancer cells Cancer Res 2005 65 11118 11128 10.1158/0008-5472.CAN-04-3841 16322262

[B39] Rowe DL Ozbay T Bender LM Nahta R Nordihydroguaiaretic acid, a cytotoxic insulin-like growth factor-I receptor/HER2 inhibitor in trastuzumab-resistant breast cancer Mol Cancer Ther 2008 7 1900 1908 10.1158/1535-7163.MCT-08-0012 18645000 PMC2586607

[B40] Camirand A Lu Y Pollak M Co-targeting HER2/ErbB2 and insulin-like growth factor-1 receptors causes synergistic inhibition of growth in HER2-overexpressing breast cancer cells Med Sci Monit 2002 8 BR521 BR526 12503030

[B41] Esparis-Ogando A Ocana A Rodriguez-Barrueco R Ferreira L Borges J Pandiella A Synergic antitumoral effect of an IGF-IR inhibitor and trastuzumab on HER2-overexpressing breast cancer cells Ann Oncol 2008 19 1860 1869 10.1093/annonc/mdn406 18641009

[B42] Jerome L Alami N Belanger S Page V Yu Q Paterson J Recombinant human insulin-like growth factor binding protein 3 inhibits growth of human epidermal growth factor receptor-2-overexpressing breast tumors and potentiates herceptin activity in vivo Cancer Res 2006 66 7245 7252 10.1158/0008-5472.CAN-05-3555 16849573

[B43] Jones RA Campbell CI Petrik JJ Moorehead RA Characterization of a novel primary mammary tumor cell line reveals that cyclin D1 is regulated by the type I insulin-like growth factor receptor Mol Cancer Res 2008 6 819 828 10.1158/1541-7786.MCR-07-2157 18505926

[B44] Gualberto A Pollak M Emerging role of insulin-like growth factor receptor inhibitors in oncology: early clinical trial results and future directions Oncogene 2009 28 3009 3021 10.1038/onc.2009.172 19581933

[B45] Ram TG Dilts CA Dziubinski ML Pierce LJ Ethier SP Insulin-like growth factor and epidermal growth factor independence in human mammary carcinoma cells with c-erbB-2 gene amplification and progressively elevated levels of tyrosine-phosphorylated p185erbB-2 Mol Carcinog 1996 15 227 238 10.1002/(SICI)1098-2744(199603)15:3<227::AID-MC8>3.0.CO;2-E 8597535

[B46] Siegel PM Dankort DL Hardy WR Muller WJ Novel activating mutations in the neu proto-oncogene involved in induction of mammary tumors Mol Cell Biol 1994 14 7068 7077 7935422 10.1128/mcb.14.11.7068-7077.1994 PMC359240

[B47] Linnerth NM Baldwin M Campbell C Brown M McGowan H Moorehead RA IGF-II induces CREB phosphorylation and cell survival in human lung cancer cells Oncogene 2005 24 7310 7319 10.1038/sj.onc.1208882 16158061

[B48] Mehrara E Forssell-Aronsson E Ahlman H Bernhardt P Specific growth rate versus doubling time for quantitative characterization of tumor growth rate Cancer Res 2007 67 3970 3975 10.1158/0008-5472.CAN-06-3822 17440113

[B49] Jones RA Campbell CI Wood GA Petrik JJ Moorehead RA Reversibility and recurrence of IGF-IR-induced mammary tumors Oncogene 2009 28 2152 2162 10.1038/onc.2009.79 19377512

[B50] Chazin VR Kaleko M Miller AD Slamon DJ Transformation mediated by the human HER-2 gene independent of the epidermal growth factor receptor Oncogene 1992 7 1859 1866 1354348

[B51] Press MF Pike MC Chazin VR Hung G Udove JA Markowicz M Her-2/neu expression in node-negative breast cancer: direct tissue quantitation by computerized image analysis and association of overexpression with increased risk of recurrent disease Cancer Res 1993 53 4960 4970 8104689

[B52] Gilcrease MZ Woodward WA Nicolas MM Corley LJ Fuller GN Esteva FJ Even low-level HER2 expression may be associated with worse outcome in node-positive breast cancer Am J Surg Pathol 2009 33 759 767 10.1097/PAS.0b013e3181b7289b 19252432 PMC3063383

[B53] Camp RL Dolled-Filhart M King BL Rimm DL Quantitative analysis of breast cancer tissue microarrays shows that both high and normal levels of HER2 expression are associated with poor outcome Cancer Res 2003 63 1445 1448 12670887

[B54] Christianson TA Doherty JK Lin YJ Ramsey EE Holmes R Keenan EJ NH2-terminally truncated HER-2/neu protein: relationship with shedding of the extracellular domain and with prognostic factors in breast cancer Cancer Res 1998 58 5123 5129 9823322

[B55] Lin YZ Clinton GM A soluble protein related to the HER-2 proto-oncogene product is released from human breast carcinoma cells Oncogene 1991 6 639 643 1674366

[B56] Pupa SM Menard S Morelli D Pozzi B De Palo G Colnaghi MI The extracellular domain of the c-erbB-2 oncoprotein is released from tumor cells by proteolytic cleavage Oncogene 1993 8 2917 2923 8105438

[B57] Scaltriti M Rojo F Ocana A Anido J Guzman M Cortes J Expression of p95HER2, a truncated form of the HER2 receptor, and response to anti-HER2 therapies in breast cancer J Natl Cancer Inst 2007 99 628 638 10.1093/jnci/djk134 17440164

[B58] Christianson TA Doherty JK Lin YJ Ramsey EE Holmes R Keenan EJ NH2-terminally truncated HER-2/neu protein: relationship with shedding of the extracellular domain and with prognostic factors in breast cancer Cancer Res 1998 58 5123 5129 9823322

[B59] Molina MA Saez R Ramsey EE Garcia-Barchino MJ Rojo F Evans AJ NH(2)-terminal truncated HER-2 protein but not full-length receptor is associated with nodal metastasis in human breast cancer Clin Cancer Res 2002 8 347 353 11839648

[B60] Saez R Molina MA Ramsey EE Rojo F Keenan EJ Albanell J p95HER-2 predicts worse outcome in patients with HER-2-positive breast cancer Clin Cancer Res 2006 12 424 431 10.1158/1078-0432.CCR-05-1807 16428482

[B61] Mehrara E Forssell-Aronsson E Ahlman H Bernhardt P Specific growth rate versus doubling time for quantitative characterization of tumor growth rate Cancer Res 2007 67 3970 3975 10.1158/0008-5472.CAN-06-3822 17440113

[B62] Bozcuk H Uslu G Pestereli E Samur M Ozdogan M Karaveli S Predictors of distant metastasis at presentation in breast cancer: a study also evaluating associations among common biological indicators Breast Cancer Res Treat 2001 68 239 248 10.1023/A:1012269804578 11727960

[B63] Tan M Yao J Yu D Overexpression of the c-erbB-2 gene enhanced intrinsic metastasis potential in human breast cancer cells without increasing their transformation abilities Cancer Res 1997 57 1199 1205 9067293

